# Association of physical activity, sedentary time, and sleep duration on the health-related quality of life of college students in Northeast China

**DOI:** 10.1186/s12955-019-1194-x

**Published:** 2019-07-16

**Authors:** Yinjian Ge, Shimeng Xin, Dechun Luan, Zhili Zou, Mengting Liu, Xue Bai, Qian Gao

**Affiliations:** 1 School of Public Health China Medical University, No.77 Puhe Road, Shenyang North New Area, Shenyang, Liaoning Province People’s Republic of China 110122; 2 Institute for Nutrition and Food Hygiene, Liaoning Provincial Center for Disease Control and Prevention, Shenyang, China

**Keywords:** Physical activity, Sedentary time, Sleep duration, Health-related quality of life, College students

## Abstract

**Background:**

College life represents a key transitional period in the life of young adults that is marked by increased social engagement; living habits acquired during this period have implications on the future life of college students. Therefore, investigation of the determinants of health status of college students is a key imperative; however, there is limited evidence on the study of concomitant effects of physical activity (PA), sedentary time (ST), and sleep duration on the health-related quality of life (HRQOL) of college students.

**Methods:**

This cross-sectional survey was conducted at a medical university in Shenyang in Northeast China in 2017. The study group comprised 926 undergraduate students. Data were collected by a self-administered questionnaire. PA, ST, sleep duration, and HRQOL were measured using the international physical activity questionnaire (IPAQ)-Long Form and the Chinese version of the 12-Item Short-Form Health Survey (SF-12). The association of PA, ST, and sleep duration with the HRQOL was examined using independent *t*-test, Pearson Chi-squared test, and multivariate linear regression analysis.

**Results:**

After adjusting for potential confounding factors, students who reported high PA had significantly higher physical component summary (PCS) score in the total study population and among female students than those who reported low PA, whereas students who reported moderate PA had significantly higher PCS score only among female students (*P* < 0.05). In the total study population and among male students, students who slept for ≥9 h/day had significantly higher mental component summary (MCS) score than those who slept for 7–< 8 h/day, whereas among only male students, those who slept for 8–< 9 h/day had significantly higher MCS score (*P* < 0.05). The interaction term between ST and PA was not statistically significant.

**Conclusions:**

PA and sufficient sleep duration had a positive impact on the HRQOL of college students; however, ST was not associated with HRQOL and there was no interaction between the impact of ST and PA on the HRQOL of college students. Increasing PA and promoting adequate sleep duration are key health promotion strategies for college students.

## Background

Health-related quality of life (HRQOL) reflects the physical and mental health as perceived by an individual or a group during a certain period. Studies have demonstrated the importance of assessing the HRQOL of the student community [1, 2], which represents a population group passing through an important phase of life. On behalf of the life development critical period university life poses great challenges and risks, which has profound effects on college students [3]. College students are known to experience a number of stressors, including change of residence [4], increased responsibility, peer pressure, and the different ways of learning and schedules [5]. Currently, the number of college students is increasing all over the world and China is no exception. For example, the total number of Chinese college students increased from 37,092 thousand in 2016 [6] to 39,809 thousand by the end of 2018 [7]. Therefore, it is meaningful to study the health status and its determinants in such a large group.

Various factors can influence the HRQOL. Several studies have shown the impact of physical activity (PA), sedentary time (ST), and sleep duration on the HRQOL [8–13]. College students tend to spend most of their time in sedentary behaviors, such as playing video games, watching movies, and online shopping [14]. A high prevalence of sedentary time was observed among college students [15]. In a global survey of 23 different income countries, between 21.9 and 80.6% of college students were physically inactive [16]. Besides, a study indicated that short sleep duration was common among Chinese college students [17]. The tendency for low PA, increasing ST, and short sleep duration among college students may have an impact on their health.

Several studies have assessed the HRQOL of the student community. Moreover, studies have demonstrated the association of either PA, ST, or sleep duration with the HRQOL of students; however, to the best of our knowledge, the concomitant effects of these three factors on the HRQOL of college students are not well characterized. One study had assessed the relation of PA, ST, and sleep duration with the HRQOL of high school students [18]; however, the manner in which these three factors affect college students and whether these factors interact with each other is not clear. The college period represents the first transitional period for young adults with respect to their social life. The living habits during this period have a great impact on their future life. Therefore, the college period may represent the last window of opportunity to comprehensively address the health problems of a large proportion of young adults. Acquisition of good habits during this period will be beneficial to their health throughout adulthood. Therefore, we performed a cross-sectional study to investigate this association among undergraduate students at a university in Northeast China.

## Methods

### Sample and design

We recruited a convenience sample of medical students attending grade 1 through 3 at a medical university located in Shenyang in Northeast China in 2017. All students who were present on the day of the study were included. Data pertaining to demographic characteristics, PA, ST, sleep duration, and HRQOL were collected using self-administered questionnaires.

A total of 1027 students were administered the questionnaire. Of these, 101 students were excluded because of various reasons. Among these, 65 students were excluded as the total daily time spent in different domains of PA was more than 16 h [19]; 36 students were excluded because of incomplete data pertaining to sleep duration (*n* = 6), body mass index (BMI, *n* = 9), or HRQOL (*n* = 21). Finally, 926 students (90.17%) were included in the analysis. Ethical clearance was provided by the ethics committee of the China Medical University.

### Demographic and other characteristics

Students self-reported their demographic characteristics, including sex, age, height, weight, grade, specialty, home location, and monthly living expenses. Some of these variables were grouped according to the research needs. The specialty was divided into two groups: clinical and non-clinical. BMI was measured as weight/height^2^ (kg/m^2^). Students were categorized as “underweight” (BMI < 18.5 kg/m^2^), “normal weight” (18.5 ≤ BMI < 24 kg/m^2^), and “overweight or obese” (BMI ≥ 24 kg/m^2^). The monthly living expenses were divided into three groups: ≤1,000 yuan/month, 1,001–1,500 yuan/month, and > 1,500 yuan/month.

### Measurement of PA, ST and sleep duration

In this study, PA, ST, and sleep duration of college students were measured using the international physical activity questionnaire (IPAQ)-Long Form [20]. The IPAQ-Long Form has already shown good reliability and validity in Chinese college students [21]. According to the classification standard [19], students were divided into three groups: low PA, moderate PA, and high PA through the questionnaire. ST was divided into four groups: < 6 h/day, 6–< 8 h/day, 8–< 10 h/day, and ≥ 10 h/day. Sleep duration was divided into four groups: < 7 h/day, 7–< 8 h/day, 8–< 9 h/day, and ≥ 9 h/day.

### Measurement of HRQOL

HRQOL of college students was assessed using the Chinese version of the 12-Item Short-Form Health Survey (SF-12), which is applicable to the general population of China [22]. The SF-12 contains 12 questions and 8 scales: physical functioning, role functioning physical, bodily pain, general health, vitality, social functioning, role functioning emotional, and mental health. Our study transformed the scores for the eight dimensions into a standard score of 0–100. Based on the scores for the eight dimensions, the physical component summary (PCS) and the mental component summary (MCS) were calculated [23]. A higher score for PCS and MCS indicated a better HRQOL.

### Statistical analysis

Descriptive statistics were calculated for the total study population as well as disaggregated by male and female subgroups. Differences in the characteristics between males and females for the continuous and categorical variables were examined using the independent *t*-test or the Pearson Chi-squared test, as appropriate. The results are presented as mean (standard deviation, SD) or frequencies (percentages, %). The statistical significance of the scores for PCS and MCS among PA, ST, sleep duration were evaluated by one-way analysis of variance. The multiplicative interaction between ST and PA was evaluated by Wald test. At the same time, the association of PA, ST, and sleep duration with PCS and MCS scores were evaluated for the total study population and male and female student subgroups using multivariate linear regression analysis. All data analyses were performed using the Statistical Package for the Social Sciences (SPSS) version 20.0. *P* values of < 0.05 were considered indicative of statistical significance.

## Results

### Characteristics of students

The characteristics of the total students stratified by sex are shown in Table 1. Of the 926 students, there were 308 (33.3%) males and 618 (66.7%) females. The average age of the students was 19.78 (1.14) years (range 17–23). Female students had significantly lower age and lower BMI than male students (age: *P* = 0.001; BMI: *P* < 0.001). Female students had higher prevalence of low PA and longer ST than male students (PA: *P* = 0.010; ST: *P* = 0.045); however, there were no significant differences with respect to sleep duration or HRQOL between male and female students.

**Table 1:** Characteristics of Chinese college students by sex ^a^

	Total (*n* = 926)	Male (*n* = 308)	Female (*n* = 618)	*P* value
Mean (SD)				
Age, years	19.78 (1.14)	19.95 (1.22)	19.69 (1.09)	0.001*
BMI^b^, kg/m^2^	21.04 (2.90)	22.14 (3.19)	20.49 (2.58)	< 0.001*
ST^c^, h	7.51 (2.66)	7.26 (2.79)	7.63 (2.59)	0.045*
Sleep duration, h	8.23 (0.84)	8.20 (0.93)	8.24 (0.79)	0.411
PCS^d^	50.00 (8.10)	50.58 (8.17)	49.71 (8.06)	0.123
MCS^e^	50.00 (9.53)	50.45 (10.30)	49.78 (9.13)	0.316
*N* (%)				
Grade				0.373
First	166 (17.9)	58 (18.8)	108 (17.5)	
Second	463 (50.0)	144 (46.8)	319 (51.6)	
Third	297 (32.1)	106 (34.4)	191 (30.9)	
Specialty				0.742
Clinical	620 (67.0)	204 (66.2)	416 (67.3)	
Non-clinical	306 (33.0)	104 (33.8)	202 (32.7)	
Home location				0.619
Urban	531 (57.3)	174 (56.4)	357 (57.8)	
Town	210 (22.7)	67 (21.8)	143 (23.1)	
Rural	185 (20.0)	67 (21.8)	118 (19.1)	
Monthly living expenses, yuan/month				0.467
≤1,000	264 (28.5)	93 (30.2)	171 (27.7)	
1,001–1,500	458 (49.5)	154 (50.0)	304 (49.2)	
> 1,500	204 (22.0)	61 (19.8)	143 (23.1)	
PA ^f^				0.010*
Low	120 (13.0)	36 (11.7)	84 (13.6)	
Moderate	369 (39.8)	105 (34.1)	264 (42.7)	
High	437 (47.2)	167 (54.2)	270 (43.7)	

^a^Values are presented as mean (SD) or N (%) when appropriate. ^b^BMI: body mass index. ^c^ST: sedentary time. ^D^PCS: physical component summary. ^e^MCS: mental component summary. ^f^ PA: physical activity

^*^P < 0.05

### Univariate analysis of PCS and MCS scores according to PA, ST, and sleep duration

PCS and MCS scores of students are displayed according to different PA groups (Table 2), ST groups (Table 3), and groups of sleep duration (Table 4) (data of Bonferroni adjustment not shown in tables). Among the total study population, the PCS score was significantly different in different PA groups (*P* = 0.003); in addition, statistically significant difference was observed in the MCS score according to sleep duration (*P* = 0.006). After Bonferroni adjustment, students in the high PA group scored significantly better than students in the low PA group with respect to the PCS score (*P* = 0.003). Students who slept for ≥9 h/day had significantly higher MCS score than those who slept for 7–< 8 h/day (*P* = 0.008). In further sex analysis, there was significant difference with respect to the PCS score of different PA subgroups of female students (*P* < 0.001) and with respect to the MCS score of different subgroups of sleep duration among male students (*P* = 0.004). After Bonferroni adjustment, the PCS score was higher with increasing PA level in females (all *P* < 0.05). Males who slept for ≥9 h/day had significantly higher MCS score than those who slept for 7–< 8 h/day (*P* = 0.003).

**Table 2:** PCS and MCS scores of Chinese college students according to different PA groups ^a^

PA ^b^	*N*	PCS ^c^	*P* value	MCS ^d^	*P* value
Total			0.003*		0.593
Low	120	47.98 (8.82)		49.23 (9.15)	
Moderate	369	49.76 (7.69)		49.98 (9.61)	
High	437	50.76 (8.16)		50.23 (9.58)	
Male			0.837		0.797
Low	36	50.61 (6.74)		50.30 (10.79)	
Moderate	105	50.95 (6.78)		50.99 (9.92)	
High	167	50.34 (9.22)		50.13 (10.47)	
Female			< 0.001*		0.366
Low	84	46.85 (9.38)		48.77 (8.38)	
Moderate	264	49.28 (7.98)		49.58 (9.47)	
High	270	51.02 (7.43)		50.29 (9.01)	

^a^Values are presented as mean (SD). ^b^ PA: physical activity. ^c^ PCS: physical component summary. ^d^ MCS: mental component summary. ^*^P < 0.05

**Table 3:** PCS and MCS scores of Chinese college students according to different ST groups ^a^

ST ^b^	N	PCS ^c^	*P* value	MCS ^d^	*P* value
Total			0.321		0.160
< 6 h/day	240	49.47 (8.91)		50.32 (8.92)	
6–< 8 h/day	291	49.66 (8.27)		50.30 (9.42)	
8–< 10 h/day	218	50.65 (7.42)		48.74 (10.40)	
≥10 h/day	177	50.47 (7.47)		50.64 (9.36)	
Male			0.452		0.607
< 6 h/day	93	49.98 (8.39)		51.27 (9.36)	
6–< 8 h/day	97	50.01 (9.04)		49.86 (10.62)	
8–< 10 h/day	61	51.32 (7.33)		49.40 (11.74)	
≥10 h/day	57	51.75 (7.06)		51.20 (9.64)	
Female			0.575		0.174
< 6 h/day	147	49.15 (9.23)		49.71 (8.61)	
6–< 8 h/day	194	49.49 (7.88)		50.52 (8.77)	
8–< 10 h/day	157	50.39 (7.46)		48.48 (9.85)	
≥10 h/day	120	49.86 (7.60)		50.37 (9.25)	

^a^ Values are presented as mean (SD). ^b^ ST: sedentary time. ^c^ PCS: physical component summary. ^d^ MCS: mental component summary

**Table 4:** PCS and MCS scores of Chinese college students according to different groups of sleep duration ^a^

Sleep duration	N	PCS ^b^	*P* value	MCS ^c^	*P* value
Total			0.732		0.006*
< 7 h/day	58	49.16 (7.65)		48.30 (10.42)	
7–< 8 h/day	274	50.35 (7.71)		48.96 (9.29)	
8–< 9 h/day	463	49.86 (8.13)		50.21 (9.33)	
≥9 h/day	131	50.12 (9.01)		52.20 (9.99)	
Male			0.531		0.004*
< 7 h/day	27	49.95 (7.92)		49.21 (10.15)	
7–< 8 h/day	87	51.68 (8.26)		47.71 (10.20)	
8–< 9 h/day	146	50.16 (8.10)		51.08 (9.73)	
≥9 h/day	48	50.22 (8.43)		54.16 (11.09)	
Female			0.828		0.296
< 7 h/day	31	48.48 (7.47)		47.50 (10.75)	
7–< 8 h/day	187	49.73 (7.39)		49.54 (8.80)	
8–< 9 h/day	317	49.73 (8.16)		49.80 (9.13)	
≥9 h/day	83	50.07 (9.37)		51.06 (9.17)	

^a^ Values are presented as mean (SD). ^b^ PCS: physical component summary. ^c^ MCS: mental component summary. ^*^P < 0.05

### Association of PA, ST, and sleep duration with PCS and MCS scores in multivariate linear regression analysis

Table 5 shows the estimated coefficients from multiple linear regression analyses after adjusting for potential confounding variables. In the total study population, students who reported high PA had significantly higher PCS score than those who reported low PA, even after adjusting for sex, age, grade, specialty, BMI, home location, monthly living expenses, and PA, ST, sleep duration, as appropriate (*β* = 2.513, *P* = 0.003). Students who slept for ≥9 h/day had significantly higher MCS score than those who slept for 7–< 8 h/day (*β* = 3.348, *P* = 0.001). In further sex analysis, males who slept for 8–< 9 h/day or ≥ 9 h/day had significantly higher MCS scores than those who slept for 7–< 8 h/day, even after adjusting for age, grade, specialty, BMI, home location, monthly living expenses, and PA, ST, sleep duration, as appropriate (8–< 9 h/day: *β* = 3.063, *P* = 0.038; ≥9 h/day: *β* = 6.986, *P* < 0.001). Females who reported moderate PA or high PA had significantly higher PCS scores than those who reported low PA (moderate PA: *β* = 2.551, *P* = 0.012; high PA: *β* = 4.153, *P* < 0.001). At the same time, the interaction term between ST and PA was not statistically significant in the total study population (PCS: *P* = 0.799; MCS: *P* = 0.626), among male students (PCS: *P* = 0.927; MCS: *P* = 0.077) or among female students (PCS: *P* = 0.794; MCS: *P* = 0.972) (data not shown in tables).

**Table 5:** Multivariate linear regression for PCS and MCS of Chinese college students

	Total ^a^				Male ^b^				Female ^b^			
	PCS ^e^		MCS ^f^		PCS		MCS		PCS		MCS	
	*β*	*P*	*β*	*P*	*β*	*P*	*β*	*P*	*β*	*P*	*β*	*P*
PA ^c^												
Low	0		0		0		0		0		0	
Moderate	1.616	0.059	0.468	0.643	0.229	0.890	−0.071	0.972	2.551	0.012*	0.432	0.709
High	2.513	0.003*	0.376	0.706	−0.484	0.753	−0.926	0.630	4.153	< 0.001*	0.822	0.483
ST ^d^												
< 6 h/day	0		0		0		0		0		0	
6–< 8 h/day	−0.036	0.961	0.171	0.841	0.448	0.717	−1.011	0.513	−0.229	0.800	1.087	0.291
8–< 10 h/day	1.230	0.117	−1.213	0.190	1.615	0.261	−2.476	0.168	1.101	0.248	−0.680	0.532
≥ 10 h/day	0.954	0.248	0.817	0.401	2.219	0.126	0.287	0.874	0.441	0.664	1.283	0.269
Sleep duration												
< 7 h/day	−1.455	0.215	−1.150	0.406	−1.353	0.464	1.219	0.598	−1.751	0.262	−2.129	0.232
7–< 8 h/day	0		0		0		0		0		0	
8–< 9 h/day	−0.533	0.389	1.322	0.070	−1.621	0.170	3.063	0.038*	−0.027	0.971	0.547	0.517
≥ 9 h/day	−0.476	0.579	3.348	0.001*	−1.683	0.270	6.986	< 0.001*	0.293	0.783	1.736	0.153

^a^ Model 1 adjusted for gender, age, grade, specialty, BMI, home location, monthly living expenses and also adjusted for PA and ST (for sleep duration) or PA and sleep duration (for ST) or ST and sleep duration (for PA)

^b^ Model 2 adjusted for age, grade, specialty, BMI, home location, monthly living expenses and also adjusted for PA and ST (for sleep duration) or PA and sleep duration (for ST) or ST and sleep duration (for PA)

^c^ PA: physical activity. ^d^ ST: sedentary time. ^e^ PCS: physical component summary. ^f^ MCS: mental component summary. ^*^P < 0.05

## Discussion

College students represent a unique group. The life habits developed during college period are possible to exert far-reaching effect on future life. Therefore, our study investigated the effects of PA, ST, and sleep duration on the HRQOL of Chinese college students.

In the current study, we found that the mean sleep duration of college students (8.2 h) was higher than that reported among adults in China (7.3 h) [24], United States (7.2 h) [25], and Korea (6.7 h) [26] or other college students from China (7.08 h) [17]. The proportion of students with sleep duration less than 7 h/day (6.3%) was lower than that reported in another study of Chinese college students (41.3%) [17]. At the same time, the mean ST of college students (7.51 h) was higher than that of middle-aged British adults (4.8 h) [27]. However, it was comparable with the mean ST of Nigerian college students (7.64 h) [15]. In our study, the prevalence of low PA (13.0%) was lower than that reported from a previous study of Chinese college students (37.3%) [16]. These differences may be because participants in our study were medical students as against non-medical students in the previous study. Studies demonstrated that medical students were apt to have better life habits than their peers due to a deeper understanding of health-related issues [28, 29]. Therefore, our study population reported longer sleep duration, longer ST, and a relatively high level of PA. We also found that females had significantly higher prevalence of low PA and higher ST than males. Similar sex-related differences were observed in previous studies [30–32]. This may be attributable to the greater propensity of male students to engage in sports activities. In our study, there were no significant sex differences with respect to sleep duration or HRQOL. However, Cherepanov et al. found clear sex difference on the HRQOL of adults in the United States [33]. A study conducted in Bhutan revealed significant sex-based difference with respect to sleep duration of adults [34]. These differences may be due to the age of participants; in our study, the age range of students was 17–23 years, which is younger than the typical adult population. Therefore, no significant difference was observed between the HRQOL of male and female students.

In our study, the results of the univariate analysis were consistent with those of multivariate linear regression analysis. In multivariate linear regression analysis, we controlled for potential confounding factors and observed the effects of PA, ST, sleep duration on the HRQOL at the same time. The results showed a positive impact of PA on the HRQOL. Pekmezovic et al. indicated that regular PA improved the HRQOL of college students [35]. Other studies have also shown a positive impact of PA on the HRQOL of Malaysian children [8], young adults in the United States [9], and civil servants in China [36]. Indeed, regular PA has many beneficial effects. PA can promote cardiorespiratory fitness [37] and reduce the risk of many diseases, such as ischemic heart disease [38], diabetes [39], and cognitive disorders [40]. To summarize, PA can not only reduce the risk of many diseases and avoid obesity, but it can also help improve the HRQOL. At the same time, we found no association between ST and the HRQOL of college students. Similar findings were reported from a study conducted in adult population from United States [41]. However, in a study by Husu et al., lower ST was associated with excellent health status of Finnish children [11]. Jalali-Farahani et al. also found that ST was associated with the HRQOL of high school students in Tehran [18]. This difference may be because our study estimated ST as the total daily time spent in sedentary behavior; we did not account for the time spent in different domains of sedentary behavior such as playing video games, watching movies, or reading. Previous studies have shown that not all domains of sedentary behavior are negatively associated with HRQOL. For example, reading time was found to be positively associated with HRQOL [10]. Therefore, more researches are needed to determine the association of different domains of ST with the HRQOL. On the other hand, our study found a positive impact of sufficient sleep duration on the HRQOL. In a study conducted on young adults in US, inadequate sleep duration (< 7 h/day) was significantly associated with low HRQOL [12]. Sithey et al. found that both short (≤6 h/day) and long sleep duration (≥11 h/day) were independently associated with poor self-reported health status of adults in Bhutan [34]. Previous studies have already shown that inappropriate sleep may be associated with elevated risk of some diseases such as diabetes, hypertension, and cardiovascular diseases [42, 43]. And irregular sleep habits are associated with increased tiredness, endocrinological, immunological, and metabolic adverse effects [44, 45]. Students who reported adequate sleep have better performance than those who reported sleep-deprived [46, 47]. In addition, short duration of sleep is related to poor health behaviors, such as drinking and smoking, which in turn increase the risk of obesity, hypercholesterolemia, and mortality [48, 49]. It seems that sleep duration is a decisive factor in people’s health. Therefore, we should promote regular PA and sufficient sleep duration as a part of strategies to improve the HRQOL of college students.

We assumed the interaction between ST and PA existed and tested it. However, the interaction term between ST and PA was not statistically significant. This showed that the effect of PA on the HRQOL of college students was independent of the effect of ST on the HRQOL. This implies that even if college students spend most time of their day in sedentary behavior, their PA can still have an independent beneficial effect on their health. This finding was similar to that of a previous study of adults in the United States, in which sedentary behavior was not associated with HRQOL, and the independent association between PA and HRQOL was not affected by sedentary behavior. Therefore, no interaction between PA and sedentary behavior was observed [41].

Our study added data on the HRQOL of college students and its determinants and examined the concomitant association of PA, ST, and sleep duration with the HRQOL of college students. However, the following limitations are acknowledged in the present study. First, our study was a cross-sectional study, which did not allow us to establish a causal relationship of PA, ST, and sleep duration with the HRQOL of college students. Thus, the causal relationship needs further longitudinal studies to verify in future. Second, our study sample comprised of freshmen, sophomores, and juniors. However, college students who are about to graduate need to complete clinical practice, graduation, and job search. Therefore, the challenges and risks they face are different from those faced by participants in our study. Therefore, their current situation and health effects may be different from our sample. Thus, our results may not be applicable to all college students. Third, some variables in our study such as ST and sleep duration were self-reported which renders them vulnerable to subjectivity. Therefore, the impact of recall and reporting bias on our results cannot be excluded.

## Conclusion

College students represent a special population that may have a variety of health problems, and these problems can influence their life and learning. Health promotion among college students is a key imperative. Our results suggest that PA and sufficient sleep duration may have a positive impact on the HRQOL of college students. However, ST was not associated with HRQOL and there was no interaction between the impact of ST and PA on the HRQOL of college students. These findings provide some novel information that may help guide future health research among college students and advance the development of specific health promotion interventions aimed at this special group. Our findings suggest that increasing PA and promoting adequate sleep duration should be a key component of health promotion strategies for college students. Our findings need to be explored and supported by further research in the future.

## Data Availability

The datasets used and/or analysed during the current study are available from the corresponding author on reasonable request.

## Abbreviations

BMI: body mass index
HRQOL: health-related quality of life
IPAQ: the international physical activity questionnaire
MCS: mental component summary
PA: physical activity
PCS: physical component summary
SF-12: the 12-Item Short-Form Health Survey
ST: sedentary time
